# Medical Items and News of Interest to the Physician

**Published:** 1879-10

**Authors:** 


### Medical Items and News,

### For the Use of Physicians.

CULLED FROM THE STANDARD MEDICAL JOURNALS OF THE WORLD.

NOTE.—These formulæ are intended only for the regular practitioner of medicine, and should be employed only by him, or under his immediate supervision.

**Pneumonia.**

Wm. Dobin, M. D., publishes in *The Practitioner*, of June, three cases in which the use of aconite in small and frequently repeated doses was evidently of service in arresting the development of pneumonia.

**Hemmorrhoids.**

C. Dradwick, M. D., has noticed the fact that in a considerable number of patients troubled with hemorrhoids who were taking valerianate of zinc for other troubles the hemorrhoids have, with few exceptions, been relieved.

**Phenylated Collodium. (Solidified Creosote.)**

℞

Cryst. carbolic acid.. } Equal parts.

Collodium.......... }

Mix. For toothache.

**Belladonna as an Intestinal Remedy.**

(P. B. Scott, M. D.) This writer considers the use of belladonna should be more frequent in painful abdominal affections of children because of its effectiveness as an anodyne and antispasmodic; its safety owing to their greater tolerance of its action, and our power to measure its effect by means of the eye and skin. He cites cases to show its efficacy in constipation, colic, perityphlitis.—*Louisville Medical News.*

**Goitre.**

Dr. Flashar reports the case of a young country girl of seventeen, who had a large exophthalmic goitre, sometimes accompanied by palpitations. He used without results the subcutaneous injections of ergotine. This remedy was then injected into the parenchyma of the goitre, and after three injections the tumor disappeared entirely, and has not returned after many years. The first injection was followed by a severe pain in the side of the neck and head, with sensation of intense heat in the head, ear, and jaw, and redness of these parts.

**Whooping-Cough treated by Tincture of Myrrh.**

(Dr. Campardon.) Whooping-cough yields rapidly to the alcholic tincture of myrrh, administered in quinine wine, or Vichy water (15 drops of the tincture to the tablespoonful.) A tablespoonful should be administered every hour or two. This treatment in no way interferes with the appropriate remedies for the tracheo-bronchitis or the pulmonary congestion.—*Bulletin Therap.*

**Hiccough.**

Dr. Grellety once saw a mother as tender, as full of affection for her children, give them a morsel of sugar dipped in table vinegar whenever immoderate or too rapid repletion of the stomach, or any other cause, had induced hiccough. The latter ceased as if by magic. Since then the Vichy physician has very frequently employed this means on his own account, and has never found it without avail.

**Acne.**

Dr. J. G. Parsons says that a far more efficacious mode of treating acne than the lotion of flowers of sulphur with glycerine and water proposed by Erasmus Wilson, is to dust the face with pure precipitated sulphur every night, using for this purpose an ordinary toilet puff. He says that this will usually effect a cure in about two weeks. If desirable, oil of lemon or rose can be added to cover the odor of the sulphur. —*British Med. Jour.*

**Poultice of Cinchona in Camphor.**

The *Restaurador Farmaceutico* recommends the following, which is officinal, in the Portuguese Pharmacopœia:

Barley flour, 2 ounces.
Powdered Loxa bark, 2 ounces.
" camphor, 1 ounce.
Water, 15 ounces.

Mix the powdered bark and the barley flour with the water, heat them till they acquire the proper consistence, and, when cold, add the camphor. The poultice is used as an antiseptic.

**Nævus.**

An instance of this is given by Dr. H. F. Sigler, in the Michigan *Medical News.* He writes: "The tumor was situated in the centre of the left cheek, and in size about as large as a dime. I procured a cork the size of the tumor, into which I inserted several fine needles, letting the points project one-eighth of an inch. I then immersed the points of the needles in pure croton oil and plunged them into the tumor. A little swelling followed, and several vesicles formed soon after. The second day a crust formed over the whole tumor. This was repeated three times, at intervals of five days, and no other treatment was required."

**Elastic Crayon of Nitrate of Silver.**

Dr. Pajot takes a laminaria-tent, two millimeters in thickness, dips it in some thick mucilage, end rolls it in finely-powdered lunar caustic. When it dries, he has a crayon, of the usual thickness of a stick of nitrate of silver, which can be introduced into the cavity of the uterus without fear of breakage. In the same manner applications can be made to other cavities, and if necessary, with stronger remedies.—*All. Med. Cent. Zeit.*

**Hypodermic Injections of Ergotine.**

Either one of the two subjoined formulas can be used :

I. Ergotine (extract of ergot) 38 grains.
Diluted alcohol, 114 minims.
Glycerine, 114 minims.

Mix. Each minim represents about one-sixth of a grain of ergotine. The dose is from five to twelve minims.

II. Ergotine, 1 drachm.
Water, 1 fluid drachm.

Dissolve and filter. Dose one to three minims or more.

**Iatraliptic Medication.**

Prof. L. P. Yandell urges the more extensive use of iatraliptic medication, that is, the rubbing into the skin of drugs and nutritious oils. He has had satisfactory results from quinia by mixing it with glycerine, ʒ j. to ℥ j., and rubbing in a sixth or a fourth of this daily. He has cured diarrhœa by rubbing tannic acid and glycerine into the abdomen, and has seen diarrhœa result from the inunction of croton oil. He has great faith in the inunction of cod-liver oil, olive oil, or hog's lard in phthisis and marasmic conditions.—*Louisville Med. News.*

**A Novel Use for Apomorphia.**

At a late meeting of the French Association for the advancement of Science, M. Verger related the case of a child with a plumb stone impacted in the gullet. No instrument being at hand for the extraction of the foreign body, recourse was had to emetics. Ipecacuanha failing to induce vomiting, it was decided, after consultation, to inject apomorphia subcutaneously. Two injections were practiced, amounting in all to two and half milligrams of the alkaloid, not a large amount. Vomiting was soon excited, and the foreign body expelled.—*The Lancet.*

**Shock.**

Dr. Charles Hunter, of the Medical School of the University of Pennsylvania, has lately introduced a new and successful mode of treatment for the great shock following railway injuries, etc. The patient is at once placed in a bath at 98°, its temperature then being rapidly raised to 110°. As is well known, the temperature of patients suffering from shock is as low as 96° in the axilla; but by this mode of treatment Dr. Hunter is enabled to raise it from 96° to 98:5°, and to reduce the number of respirations from 36 to 20. Prior to the bath the skin is cold and clammy; but on taking the patient out of it afterten or fifteen minutes, the skin is warm and dry.—*New York Med. Record.*

**Epilepsey.**

Female, aged 18, subject to epilepsy since 12 years of age, in the form of attacks of *grand mal* at monthly intervals, increasing in frequency and intensity after puberty. A formula identical with that known "Brown-Sequard's," but having sodium substituted for the potassium salt, was used, containing a dose of twenty grains thrice daily, accompanied with half a drachm of Squibb's fluid extract of ergot. The medicine was continued for eighteen months and the attacks ceased after the first four months. At the end of three years and ten months there has been no return of the disease.—*St. Louis Courier of Med.*

**Cystitis.**—(Dr. Wm. Gross.)

℞

Copaibæ, 4 gm.

Acidi benzoici, 2 gm.

Acaciæ pulv. 8 gm.

Sacchari pulv. 8 gm.

Aquæ Camphoræ, 200 gm.

Olei gaultheriæ, 29 gtt.

S. A tablespoonful to be taken every five hours, when the inflammatory symtoms have subsided. The bladder is at the same time to be washed out with tepid water, to which may be added one grain of potassium permanganate for each fl. oz. of water, if the urine has a fetid smell.

---

**Grindella Robusta in Whooping-Cough.**

At a recent meeting of the Suffolk District Medical Society, Dr. Pattee called attention to the beneficial effects of the drug in certain pulmonary affections, and remarked that *most of the fluid extracts sold in this market was said to be worthless.* Dr. Pattee had used the tincture in bronchitis, asthma and whooping-cough, in doses of half a drachm or more, repeated every one or two hours. The effect was said to have been curative in thirty cases of whooping-cough, after three or four days, without the occurrence of relapses. The dose for a child two years old would be about ten drops.—*The Pharmacist.*

---

**Cardiac Dropsy.**

Dr. Shapter, in an article on this subject in *London Practitioner*, concludes as follows: Caffein, similarly to belladonna, appears to increase vascular pressure, and consequently promotes cardiac contraction through the medium of the splanchnic nerves. Whatever may be the true theory of the action of citrate of caffein, the opinion is practically forced upon me through marked success in certain cases of the same type, and equally marked failure in other cases of a different type, that the caffein in doses of from gr. iij. to gr. vj. is a diuretic and cardiac stimulant of great value in such cases of cardiac dropsy where a dilated, feeble, and irregularly contracting heart undergoing progressive mural decay is the main clinical and pathological element to be contended against.

---

**Retention of Urine.**

Dr. Tidd relates a case of twenty-four hours retention of urine in a young woman in the eighth month of pregnancy. On account of tumefaction of the genital organs and some deviation of the urethra, all attempts at catheterism had failed; morphia was administered and punctures of the bladder proposed, when Dr. Tidd remembered the success which had attended the administration of chloral in like cases in the hands of some surgeons. He prescribed a solution of 10 grammes (150 grains) of chloral in 60 grammes (℥ ij.) of water, and administered it in teaspoonful doses at first every half-hour, and then every two hours. Deep sleep ensued, in which the patient unconsciously passed an enormous quantity of urine. Excretion commenced five minutes after the second dose of the solution. Seven days later natural labor occurred; child living and healthy; no recurrence of retention.—*Archives Med. Belg. Gaz. Hebd.*

---

**Epilepsy.**

The bromides were thought at one time to exercise a powerfully curative action in this terrible disease, but Dr. Kunze, of Halle, says he has tried the bromides enough; they only check the attacks for a few months, when they return more severe then ever. Woorara he has tried in eighty cases, of which he believes six to have been radically cured. He uses the woorara thus:

℞

Wooraræ, 0.3.

Aquæ destil, 5.0.

Mucilaginis, gtt. 1-2.

For six or eight injections, one every five days. The treatment is then stopped until the fits recur, when it is recommenced.

---

**Hamilton's Tonic.**

℞

Strychniæ sulph, gr. viij.
Cinchonidiæ sulph. ℥ i.
Tr. ferri chlor. ℥ vi.
Syr. zingiberis.
Acid. phosphoric. dil. *aa* ℥xvi,.

Dose, 1 teaspoonful, t. i. d.

VANCE'S TONIC MIXTURE (Dr. Winston).

℞

Strychniæ sulph. gr. i.

Quiniæ sulph. ʒ i.

Ferri pyrophos, ʒ i.

Pot. brom. ʒ ij.

Syr. zingiber.

Aquæ, *aa* ℥ ij. M.

Dose, one to two teaspoonfuls, t. i. d.

---

**Sweating.**

Dr. C. P. Carpenter, in a communication to the *Medical Brief*, says: In the winter of 1878, I was attending a medical friend, who was at the time, confined to his bed with a severe attack of intercostal rheumatism, which continued for seven weeks. After the acute stage passed the patient was much prostrated, and convalescence retarded by profuse sweating during sleep, which symptoms resisted some half dozen or more of the usual remedies. At the suggestion of the patient, I administered one evening, two full doses of chloral hydrate, with the view only of obtaining "one night's sound sleep at any cost." No sooner was the patient completely under the influence of the drug than the sweating ceased and returned no more, the patient making a rapid recovery. Impressed with the idea that I had *blundered* on a good thing, I have put it to the test in a number of obstinate cases of night-sweats, and with uniform success.

---

**Enruesis.**

Dr. J. C. Flood recommends the employment of tincture of cantharides in doses of a minim, and tincture of the chloride of iron given three times daily in gradually increased doses. The following is suggested by Dr. Holderness.

℞

Acid. Benzoici, ʒ ij.

Syr. aurantii, ʒij.

Aquæ, ad fl ℥ ij.

A half-ounce to be given three times daily, and the third dose to be taken after emptying the bladder and going to bed.

Another formula is as follows :
Potassii bromidi, ℥ i.
Ext. belladonæ, gr. iv ad vi.
Infus. digitals, ad fl, ℥ viij.

For an adult half an ounce twice daily ; for a child one drachm, three times daily.

---

**Acute Sciatica and Facial Neuralgia.**

A man liable to rheumatism had sciatica, due to exposure while boating. Morphia hypodermically and by the mouth, and bromide of potassium and chloral fail to do more than mitigate the pain. Doses of eight grains of salicylic acid dissolved in solution of acetate of ammonium, at intervals of two hours, produced entire relief while they were continued, and they were afterwards increased to 12 and 16 grains. Convalesence was aided with sulphate of cinchonidia and Blancard's pills. A second case, without a history of rheumatism, occured in a lady who had speedy relief with the same treatment. Still a third case was also cured by doses of 12 grains of salicylate of sodium.

A woman suffering with facial neuralgia, who was unrelieved by sulphate of cinchonidia and guarana, had immediate relief of pain following one dose of 16 grains, and continued to take the remedy in 4-grain doses (to avoid nausea) until evening. Two other cases of similar nature were in like manner cured by doses of 10 to 12 grains of salicylate of sodium repeated every two hours, marked relief following the first dose.

---

**Diarrhœa Mixture.**

It is not possible to recommend a formula which would be applicable in all cases. There are various preparations in common use, as remedies in simple diarrhœa accompanied by tenesmus, colicky pains, etc., among which Dr. Squibb's Cholera Mixture, and Velpeau's Diarrhœa Mixture are probably the best :

DR. SQUIBB'S CHOLERA MIXTURE.

℞
Tinct. opii,
" capsici,
Spr. camphoræ, āā fl. ℥ i.
Chloroformi purificati, fl. ʒ iij.
Alcoholis, q. s. ad fl. ℥ v. M.
Dose for an adult : 20 to 40 minims.

VELPEAU'S DIARRHŒA MIXTURE :

℞

Tinct. opii.

" rhei,

" opii camphoratæ, āā fl. ℥ i.

Spts. menthæ. piperitæ, fl. ʒ x.

Tinct. capsici, fl. ʒ vi.

The following mixture, which is less acrid and biting, will also be found generally useful :

℞
Tinct. kino,
" krameriæ, āā fl. ℥ i.
Syr. krameriæ,
" rhei aromatici āā fl. ℥ ss.
Tr. zingiberis, fl. ʒ i. M

---

**Epithelioma.**

Dr. John B. Rice, of Fremont, Ohio, reports in the *Michigan Medical News*, a case with the following characteristics : "A small ulcer situated on the gums in front of the middle incisors. Not at this time painful, but annoying to the patient. At first it appeared like a small blood blister." This is the history as given by the patient. When seen by Dr. Rice, "the edges were thickened, and an excavation, central, sufficiently large to cover a grain of corn, surrounding tissues normal, general health of patient good. Under local treatment the ulcer increased, gradually enlarged, and emitted a suspicious fetor. After four months it had so increased as to involve the gums both in front and behind the incisors, (these becoming loose), as well as all the tissues, quite through to the integument covering the chin. Borders of the ulcer were thickened and the sublingual glands considerably enlarged and of scirrhous hardness. The patient was seen by other surgeons. They were decided that this was a case of epithelioma. We must also say a clear case.—About this time, at the suggestion of Dr. A. C. Miller, thuja occidentalis, (arbor vitæ) was tried. Dr. M. had used the thuja with encouraging results ; was led to use it because of favorable reports from Dr. J. R. Leaming, of New York. The patient took 14 drachms of a saturated tincture daily, using also as a local application a strong infusion of the same. Improvement began soon and was gradual. The tumefied glands grew softer and diminished in size, the ulcer assumed a more benign appearance, as did also the parts thickened by infiltration, a complete cure being established in about four months after beginning the thuja.

"Dr. Leaming has used this agent for several years, using the fluid extract or saturated tincture. Beginning with drachm doses, it may be given for pulmonary hæmorrhage, as well as for cancerous ulcerations or tumors, and locally may be applied to the cavity, in the os, or to the cervix of the uterus in malignant diseases, or in non-malignant when there is a flabby condition of the parts, with a tendency to bleed; and also under the same conditions to the throat."

---

**Koumiss.**

Dr. Jas. Thompson briefly reports the following cases in the *British Medical Journal:*

*Case* 1.—E. G., a lady, aged 45, bedridden for the last five years with an abdominal tumor, suffered much from sickness, retaining food with difficulty. She gradually* wasted extremely, koumiss was prescribed, a wineglassful every two hours. This was retained without discomfort, and for several days no other food was given ; then small quantities of farinaceous food were added, and a marked improvement took place in her appearance, which continued to improve until she became better than she had been for years before.

*Case* 2.—S. S. S.,aged 28, had phthisis of several years duration, and was much reduced by copius hæmoptysis and hemorrhoids, bleeding freely. He derived the greatest benefit from a bottle of koumiss daily, and soon improved in weight, and is now more than twenty pounds heavier than he was before.

*Case* 3.—H. B., aged 22, suffers from severe dyspepsia, often dreading to eat anything. During this last term he was at Oxford college, where the food is often of an inferior description. In ten weeks he lost two stones in weight and was reduced to a skeleton. I advised the use of koumiss and a good digestible diet, and in ten days he increased seven pounds in weight ; the improvement gradually increased, at the rate of four pounds a week, and at the same time, great improvement in the dyspepsia. He continues to improve, and will soon be as well as before.

*Case* 4—A. F., aged 23, was confined to the sofa by a spinal disease, and could not eat anything. Koumiss was prescribed some time since with great benefit, and her improvement was most marked ; she almost lives on it, and if she miss a day she falls off appreciably in appearance.

---

**Colds.**

Horace Dobell, in his little work on "Coughs, Colds and Consumption," gives the following plan for stopping a cold. If employed sufficiently early it is said to be almost infallible: 1. Give five grains of ses-carb. of ammonia and five minims of liquor morphine in an ounce of almond emulsion every three hours. 2. At night give ℥ jss. of liq. ammon. acetatis in a tumbler of cold water, after the patient has got into bed and been covered with several extra blankets. Cold water should be drunk freely during the night should the patient be thirsty. 3. In the morning the extra blankets should be removed so as to allow the skin to cool down before getting up. 4. Let him get up as usual and take his usual diet, but continue the ammonia and morphia mixture every four hours. 5. At bedtime the second night give a compound colocynth pill. No more than twelve doses of the mixture from first to last need be taken as a rule; but should the catarrh seem disposed to come back after leaving off the medicine for a day, another six doses may be taken and another pill. During the treatment the patient should live a little better than usual, and on leaving it off should take an extra glass of wine for a day or two.—*Michigan Med. News.*

**Anti-Rheumatic Mixture.**

(Mistura Antiarthritica.)

℞

Potasii iodidi, ʒ v.

Vini colchici sem., ℥ i.

Tr. cimicifugæ rac., ℥ ij.

Tr. stramon, ℥ ss.

Tr. opii camph., ℥ iss. M.

Doses ʒ i, three times a day.

LA FAYETTE MIXTURE.

℞

Bals. copaivæ.
Spts. ether, nit.
Spts. lavand. co. *aa* ℥ iv.
Liquor. potassæ, ʒ iv.
Mucilag. acac., ad. Oij.

MIXTURE OF MERCURY AND IODIDE OF POTASS.

(Dr. Abbe.)

℞

Hydrag. biniodidi, gr. viij.
Potassii iodidi, ʒ xx.
Aquæ,
Syr. sasaparill. co., *aa* ℥ viij. M.

MIXTURE OF MERCURY AND IODIDE OF POTASS.

(Dr. Bulkley's.)

I. Hydrargyri bichloridi, gr. i.

Potassi iodidi, ʒ iv.

Ferri et ammon. citr., ʒ i.

Aquæ, ad. *f.* ℥ i.

II. Tinct. nucis vomicæ, ʒ ij.

Tinct. cinchon. co., ad., ℥ iij.

Mix No. 1 and No 2 at time of dispensing, and label *shake.*

Dose, ʒ i. t. i. d.

---

**Diphtheria.**

In the first place, as diphtheria is a contagious disease, and under certain circumstances not entirely known, very highly so, it is important that all practical means should be taken to separate the sick from the well. As it is also infectious, woolen clothes, carpets, curtains, hangings, etc., should be avoided in the sick room, and only such materials used as can be readily washed.

All clothes, when removed from the patient, should be at once placed in hot water. Pocket handkerchiefs should be laid aside, and in their stead soft pieces of linen or cotton cloth should be used, and at once burned.

Disinfectants should always be placed in the vessels containing the expectoration, and may be used somewhat freely in the sick room; those being especially useful which destroy bad odors without causing others (nitrate of lead, chloride of zinc, etc.) In schools there should be especial supervision, as the disease is often so mild in its early stages as not to attract common attention; and no child should be allowed to attend school from an infected house, until allowed to do so by a competent physician. In the case of young children, all reasonable care should be taken to prevent undue exposure to the cold.

Pure water for drinking should be used, avoiding contaminated sources of supply; ventilation should be insisted upon, and local drainage must be carefully attended to. Privies and cesspools, where they exist, should be frequently emptied and disinfected; slop water should not be allowed to soak into the surface of the ground near dwelling houses, and the cellars should be kept dry and sweet. In cities especially in tidal districts, basin, baths, etc., as now connected with drains, should never communicate directly with sleeping rooms.

In all cases of diphtheria fully as great care should be taken in disinfecting the sick room, after use, as in scarlet fever. After a death from diphtheria, the clothing disused should be burned or exposed to nearly or quite a heat of boiling water; the body should be placed as early as practicable in the coffin, with disinfectants, and the coffin should be tightly closed. Children, at least, and better adults also in most cases, should not attend a funeral from a house in which a death from diphtheria has occurred. But with suitable precautions, it is not necessary that the funeral should be private, provided the corpse be not in any way exposed.

Although it is not at present possible to remove at once all sources of epidemic disease, yet the frequent visitation of such disease, and especially its continued prevalence, may be taken as sufficient evidence of insanitary surroundings, and of sources of sickness to a certain extent preventable.

It should be distinctly understood that no amount of artificial "disinfection" can ever take the place of pure air, good water, and proper drainage, which cannot be gained without prompt and efficient removal of all filth, whether from slaughter houses, etc., public buildings, crowded tenaments, or private residences.—*From the Massachusetts State Board of Health.*

---

**Trousseau's Poultice for Rheumatic Arthritis.**

Dr. Dieulafoy, in *Paris Medical*, gives the following directions for making and applying the poultice in question, which he recommends as excellent for preventing cases of inflammation of the joints from passing into the chronic condition:—

Take, according to the size of the articulation, from one kilogramme and a half (52 ounces) to two kilogrammes (70 ounces) of bread. Two kilogrammes are required for the knee-joint, and one is sufficient for the wrist. Cut the bread into pieces, removing the hard portions of the crust, and steep it in water for about a quarter of an hour. On removing it from the water the bread is found to be well saturated with water, and is placed in a cloth or napkin, and the excess of water is pressed out so as to leave it merely moist.

The bread thus prepared is set for three hours on a water-bath, and now forms a sort of half-dried paste, which is softened by the gradual addition of spirit of camphor. The mass is now kneaded for about five minutes, or until it acquires the tolerably firm consistence of plum-pudding or glazier's putty. Here, indeed, is the delicate point of the manipulation, the right degree of consistence being essential ; for if the poultice is too soft it spreads out under the pressure to be applied on the articulation, while, if it is too hard, it is no longer homogeneous, it crumbles and the dry pieces may injure the skin. Hence it is necessary to carefully watch the degree of consistency of the mass. The usual tendency of those unaccustomed to the preparation is to make it too soft, through either insufficient pressure before the bread is set on the water-bath, or too rapid and too copious addition of the camphorated alcohol.

The paste thus obtained is to be spread on a piece of linen cloth of the shape of an elongated rectangle, and of a size sufficient to cover the whole joint. It is desirable that near the edges the poultice be at least of a centimetre (two-fifths of an inch) in thickness, so as to avoid the too rapid desiccation to which thin edges are liable. Over the surface of the cataplasm the following mixture, made very liquid, is spread :

Camphor, 110 grains.
Aqueous extract of opium, 77 grains.
Extract of belladonna, 77 grains.
Alcohol, sufficient.

The poultice being ready, its application is very simple. It is placed direct on the skin and covered with oiled silk to prevent evaporation. The whole is set in place by means of moderately strong pressure, exerted with a flannel bandage several yards long. Lastly, a second bandage of linen cloth of the same length is tied over in the same manner.

Thus bandaged, the articulation becomes immovable and forced to complete rest. The compression exerted must be rather strong, not enough so, however, to produce œdema. The poultice is to remain in place eight or ten days, and, on being removed, it it found as fresh and moist as when first put on, the skin being in a healthy state.

Trousseau's cataplasm is indicated in the case of a scrofulous and lymphatic patients, persons having gonorrhœa, and women in pregnancy, where arthritis has a greater tendency to assume a chronic form than under ordinary circumstances.

---

This being the season of year when people are most liable to "colds and coughs, we present the following:—

**Cough Mixtures.**

EMULSION OF COD LIVER OIL WITH SOLUTION OF SACCHARATED LIME.
(75 per cent. emulsion.)

℞

Ol. morrhuæ, ℥ vi.

Ol. anisi. ʒ ss.

Ol. sassafras, gtt. x.

Liquor calc. sacchar, ℥ ii. M.

Not compatible with acids.

EMULSION OF COD LIVER OIL AND GLYCERINE.

℞
Ol. morrhuæ, ℥ viii.
Glyconini, *f.* ℥ ii.
Aqæ amygdal. amar.
Aquæ cinnamomi, *aa* ℥ iii. M.

COD LIVER OIL MIXTURE (Dr. Wheelock).

℞

Syr. ferri iodidi, ℥ ss.

Ol. morrhuæ, ℥ iiiss. M.

Dose, two to four drachms.

EMULSION OF COD LIVER OIL WITH IRON (Dr. Knapp).

℞
Emulsion of cod liver oil, ℥ viii.
Dialyzed iron, ʒ vi. M.

Dose, one tablespoonful.

STOKES' EXPECTORANT MIXTURE.

℞
Ammon. carb., gr. viii.
Tinct. scillæ, ʒ ii.
Syr. senegæ, ℥ ss.
Tr. opii camph., ℥ i.
Aquæ, ad. ℥ iv.

WARD COUGH MIXTURE (Dr. Chambers).

℞

Fld. ext. pruni virg., ℥ iii.
Sol. potassii cyanid. gr. viii.
Sol. morphiæ Magendie, ℥ ss.
Syr. simplicis, ℥ x.
Aquæ, ℥ xviii.

Dose, a teaspoonful p. r. n.

---

The following Ointments are dispensed in the New York Hospital:

CARBOLIZED VASELINE (Saturated).

℞

Vaseline, ℥ xx.
Acid carbolic crystal, ℥ i.

Melt each separately and mix.

OINTMENT OF CHRYSOPHANIC ACID, CONCENTRATED (Dr. Bulkley).

℞

Acid chrysophanic, ℥ i.
Ung. simplex, ℥ iv.

Melt the ointment, and while hot add the acid, stirring till dissolved.

BROWN OINTMENT.

℞

Pulv. acid salicylic, gr. xl.

Bals. Peruvian, ʒ i.

Vaselinæ, ℥ i.

OINTMENT OF SALICYLIC ACID.

℞

Pulv. acid salicylic, ʒ i.

Vaseline, ℥ i.

OINTMENT OF IODOFORM.

℞

Iodoform, ʒ i.

Vaseline, ℥ i.

Reduce the iodoform to powder and add to the vaseline; heat by water bath till disolved.

OINTMENT OF PERUVIAN BALSAM (Dr. Morris).

℞

Bals. Peru., ʒ ii.

Cerat. simplic., ℥ i. M.

---

**Chlorate of Potassa Mixture.**

℞

Ammon. muriat.

Potass. chlorat., *aa* ʒ i.

Ext. glycyrrh, ʒ ss.

Aquæ cinanam., ad. ℥ iv.

Dose, a tablespoonful.

MIXTURE OF IODIDE OF POTASSIUM AND HOFFMAN'S ANODYNE.

℞

Potass. iodid. ʒ iii.

Spts. ether. co., ℥ i.

Syr. pruni. virg., ℥ iii. M.

Dose a teaspoonful.

WARD BELLADONNA LINIMENT (Dr. T. R. Chambers).

℞

Ext. belladonæ fl., ℥ ii.
Tr. aconiti rad., ℥ i.
Chloroform, ℥ i.
Spts. camphoræ, ℥ iv.
Alcohol dilut. ad., O ii.

STOKES' LINIMENT.

℞

Ol. terebinth. ℥ iii.

Acid. acetic fort. ℥ ss.

Ol. Limonis. ʒ i.

Vitell. ovi. No. i.

Aquæ, ℥ iii. M.

COMPOUND IODINE LINIMENT.

℞

Iodini, gr. ii.

Camphor, ʒ ss.

Ol. lavand.

Ol. rosemar. *aa* ♏ xv.

Alcohol, *f.* ℥ iss.

Aquæ amoniæ, ad. ℥ ii.

HYPODERMIC SOLUTIONS.

EXT. ERGOT SOLUTION.

℞

Ext. ergot (Squibb's) 1 part.
Carbolized distilled water, 5 parts.
(Carbolized distilled water 1-1000.)

MAGENDIE'S SOL. MORPHIA.

℞

Morphiæ sulph. gr. lxxx.
Distilled water (carbol.) f ℥ v.

M. and filted.

LENTE'S SOLUTION OF QUINIA.

℞

Sulph. quiniæ, gr. lxxx.
Acid. sulph. dil., q. s.
Aquæ, ad. f. ℥ i.

Heat to boiling and add.

Acid. carbolic, gr. v.

SOL. PILOCARPIA MURIATE.

℞

Pilocarpiæ mur. gr. i.
Aquæ dest. (carbol.) ♏ l.

Dose, ten minims.

SOL. APOMORPHIA MURIATE.

℞.

Apomorphiæ muriat. cryst. gr. i.
Aquæ dest. ♏ lxxx.

Dose, 10 ♏ for emetic.

To be prepared only at the time it is wanted.

